# The Effects of Static Stretching Intensity on Range of Motion and Strength: A Systematic Review

**DOI:** 10.3390/jfmk8020037

**Published:** 2023-03-24

**Authors:** Joseph Bryant, Darren J. Cooper, Derek M. Peters, Matthew David Cook

**Affiliations:** 1 School of Sport and Exercise Science, University of Worcester, Henwick Grove, Worcester WR2 6AJ, UK; 2 School of Allied Health and Community, University of Worcester, Henwick Grove, Worcester WR2 6AJ, UK

**Keywords:** stretch intensity, stretching, flexibility, strength

## Abstract

The aim of this study was to systematically review the evidence on the outcomes of using different intensities of static stretching on range of motion (ROM) and strength. PubMed, Web of Science and Cochrane controlled trials databases were searched between October 2021 and February 2022 for studies that examined the effects of different static stretching intensities on range of motion and strength. Out of 6285 identified records, 18 studies were included in the review. Sixteen studies examined outcomes on ROM and four on strength (two studies included outcomes on both ROM and strength). All studies demonstrated that static stretching increased ROM; however, eight studies demonstrated that higher static stretching intensities led to larger increases in ROM. Two of the four studies demonstrated that strength decreased more following higher intensity stretching versus lower intensity stretching. It appears that higher intensity static stretching above the point of discomfort and pain may lead to greater increases in ROM, but further research is needed to confirm this. It is unclear if high-intensity static stretching leads to a larger acute decrease in strength than lower intensity static stretching.

## 1. Introduction

Static stretching is a common technique purported to improve range of motion (ROM) within sporting and rehabilitation settings and it is a passive lengthening of a muscle which is then sustained [1]. The increase in ROM results from an increase in stretch tolerance and a decrease in passive stiffness of the musculotendinous unit (MTU) [2]. Completed before exercise, the effects on performance are unclear, with some studies observing positive effects [3] and others impaired performance [4]. As a result, the European College of Sports Sciences [5] and the American College of Sports Medicine [6] do not recommend the use of static stretching and instead promote dynamic stretching. Despite these recommendations, static stretching is still used in warm-ups and promoted by coaches [7,8,9,10]. 

Good ROM, or flexibility around a joint, is important for the performance and activities of daily living as it allows full usage of the functional range. There are also suggestions that poor flexibility [11] and higher MTU stiffness [12] are associated with a greater risk of muscular injury. This occurs because demands in energy absorption and release may rapidly exceed the stiffness capacity of the MTU [13]. As a result, there is a need to balance the associated benefits of increasing ROM to decrease injury risk, against reducing performance from a level of stiffness that contributes to force production during dynamic movements where the MTU stiffness is important. Furthermore, this balance is made following limited research on the intensity of stretching as a variable.

Four variables can impact the effectiveness of stretching: frequency, duration, the position held and the intensity [14,15]. Whilst the duration and frequency of stretching are simple for participants to understand and implement, the intensity of stretch and position held for each stretch is far more subjective. Due to their inherently subjective nature, the effects of different stretch intensities and positions on ROM and exercise performance are harder to control and research. 

Stretching intensity does not have a single definition. Jacobs and Sciascia [16] defined it as “the magnitude of force or torque applied to the joint during a stretching exercise”, however, Freitas et al. [17] defined it as “degree of muscle-tendon lengthening induced by a change in joint range of motion that is controlled by an individual’s subjective tolerance to stretch”. Historical recommendations for stretching intensity were to elicit the maximal ROM without pain or discomfort [18]. However, subsequent investigations have then examined the influence of stretching intensity on ROM, with observations demonstrating stretching to a higher intensity (120% point of pain) compared to a lower intensity (80% with no pain) elicited a greater change in ROM [19]. Therefore, intensity may be an important consideration in eliciting greater changes in ROM. However, it is important to recognise that these investigations have limited ecological validity to athletic populations and practice within an applied environment, where stretching is included within warm-up routines.

Static stretching has been observed to decrease strength immediately following the stretching bout [20,21]. This is potentially caused by a more compliant MTU, a less stiff MTU [22] and lower motor unit activation [23]. Furthermore, the intensity of static stretching on strength has also been investigated with findings of both greater decreases in subsequent strength [19,24] and no changes [25,26], therefore the intensity of stretching may also be important on subsequent strength. 

Stretching intensity is subjective, with studies investigating stretching intensity using the participant’s perception of the intensity, often using the terms ‘point of pain’ or ‘point of discomfort’ [19] or a numerical rating scale (NRS) for pain or discomfort (0 = no pain, 10 = worst imaginable discomfort or pain) [27]. As a result, this variability in methods makes stretching at different intensities difficult to define and implement within an applied setting. Furthermore, the outcomes are unclear and therefore it is difficult to determine if high-intensity stretching is more beneficial to increase ROM and strength. Multiple studies have examined the effects of intensity of static stretching on ROM and to the authors’ knowledge, no previous studies have systematically collated these together to identify if the intensity of static stretching is important for eliciting changes in ROM and strength. There is also a lack of systematic reviews on this topic and practical recommendations that contribute to the understanding on the effects of intensity on static stretching on subsequent range of motion and strength. Therefore, this study aimed to systematically review the effects of different static stretching intensities on ROM and strength.

## 2. Materials and Methods

### 2.1. Ethical Approval

The study was approved by the College of Business, Psychology and Sport Research Ethics Panel (Reference: CBPS21220019) and performed at the University of Worcester, UK.

### 2.2. Literature Search Strategy 

An electronic database search was conducted of PubMed, Web of Science and Cochrane controlled trials databases between October 2021 and February 2022 and a follow-up search in March 2023. The following search terms were used to find relevant publications: “static stretching”, “static stretching intensity”, “exercise performance”, “range of motion”, “ROM”, “flexibility”, and “strength”. The search protocol used Boolean operators and required the title, abstract or keywords to include the search terms (“static stretching OR static stretching intensity) AND (“range of motion”, OR “ROM”, “flexibility”, OR “strength”).

For the search strategy, static stretching was defined as “taking one or multiple joints to the maximum range of motion the individual can tolerate as the muscle lengthens and is held in that position for 20 s or more.” To encompass multiple methods of inducing different intensities of stretching, static stretching intensity was defined as the “magnitude of force or torque applied to the joint during a stretching exercise [16]” and the “degree of muscle-tendon lengthening induced by a change in joint range of motion that is controlled by an individual’s subjective tolerance to stretch [17]”. The follow-up search added one additional article to the review. 

### 2.3. Inclusion and Exclusion Criteria

The inclusion criteria were as follows: (1) participants: adults aged 18–50 with no history of serious injury or an ongoing injury, non-athletic and athlete population; (2) intervention: static stretching with the intensity variable measured; (3) comparisons: pre- or post-comparison of experimental conditions with static stretching intensity as an independent variable (e.g., high-intensity versus low-intensity static stretching); (4) outcomes; range of motion as measured by degrees (°) or flexibility or strength; (5) study design; randomised controlled trials, non-randomised controlled trials, parallel-group designs or single- and double-blinded to outcomes. 

**Figure 1:**
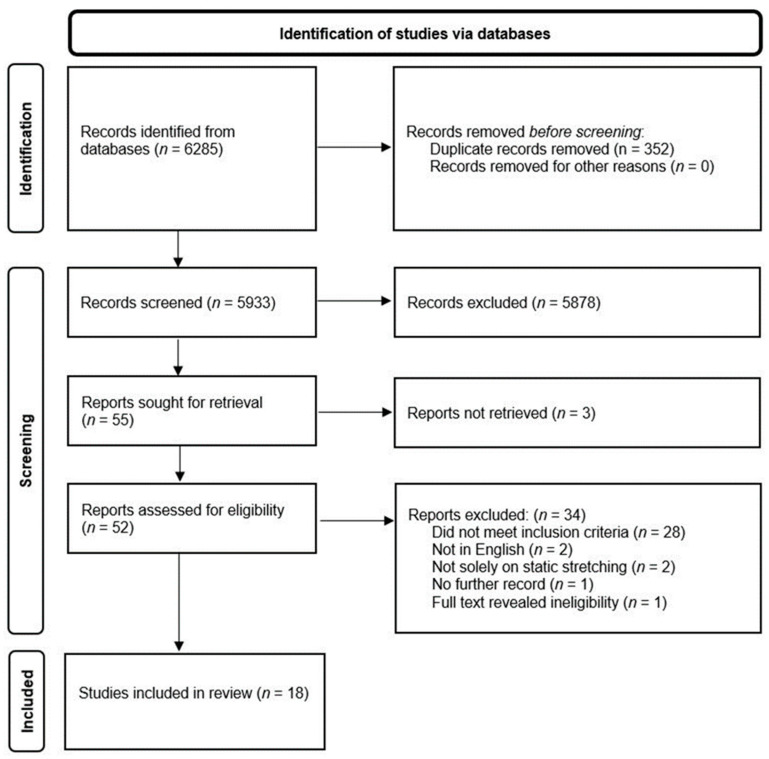
The systematic process for including studies within the review.

Studies were excluded if: they used injured participants; looked at the effects on injury prevention; intensity was not an independent variable; and no performance measures were used such as ROM or muscle force and strength. Studies examining passive stiffness or shear modulus following static stretching of different intensities were also excluded. No systematic reviews, meta-analyses, letters to the editors, book chapter and conference abstracts were included in this study. There were no limitations on joint angle, frequency, volume, or types of contraction, as it would have excessively narrowed the searches. In addition, the review did not limit the duration of the intervention (i.e., acute, or chronic) as it was primarily concerned with outcomes from static stretching at different intensities. The review utilised the guidelines for Preferred Reporting Items for Systematic Review and Meta-Analyses (PRISMA) [28] (Figure 1).

### 2.4. Study Selection

Immediately following the exclusion of duplicates, study titles and abstracts were independently screened by two authors to determine relevant studies. Disagreements between the two authors screening the articles were discussed and resolved by a third author. 

### 2.5. Data Extraction

The studies then underwent detailed analysis by the lead and last author to be included in the review. Studies with no data available, clinical trial registration, or data presented within conference proceedings were excluded from the review. The lead and last authors extracted the following information from the included studies: authors, date of publication, sample size (n), study design, participant characteristics (age, training status), stretching intervention, outcome measures (range of motion and strength) and results. 

### 2.6. Quality Assessment 

Risk of bias was assessed using the PEDro tool (https://pedro.org.au/english/resources/pedro-scale/ (1 November 2021)). The scales contain 11 items, of which 10 are scored as a Yes or No (i.e., 1 or 0). Item one, which questions eligibility criteria, identifies external validity, so it does not form the overall PEDro score. Studies with a PEDro score of 0 to 3 points were deemed “low quality”, 4 to 5 points as “moderate quality” and scores of 6 to 10 points as “high quality”. 

## 3. Results

The database searches yielded 6285 articles of which 352 were then removed due to duplication. A subsequent 5878 articles were then excluded based on their titles and abstracts alone. The full texts of 52 articles were subsequently retrieved of which 18 studies were included in the review for meeting the inclusion criteria (Figure 1).

### 3.1. Studies Examining Effects of Static Stretching Intensities on Range of Motion

Sixteen studies examining static stretching intensities on range of motion are summarised in Table 1. These studies had a total of n = 316 participants, of which n = 260 were men and n = 56 were women. 

All the studies included in the review found that static stretching increased ROM. However, eight studies demonstrated that higher intensity static stretching elicited greater changes in ROM [19,26,29,30,31,32,37,41] and the other eight studies demonstrated there was no benefit of higher intensity stretching to increase ROM [27,33,34,35,36,38,39,40]. 

The studies used a variety of different static stretching protocols, this included a percentage of pre-intervention ROM [37]; NRS [27]; verbal rating scale [36,41]; stretching to a point of discomfort and an added percentage past this point [19,26,32,33,35,38,39]; stretching at and past a ROM without pain [31], a percentage of the maximum passive resistive torque [34] and a percentage of maximum tolerated stretch torque without pain [30]. One study examined the cross-education effect of stretching on the dominant leg and then observed effects on the non-dominant leg [41]. 

### 3.2. Studies Examining the Effects of Static Stretching Intensities on Strength 

Four studies examined the effects of static stretching intensities on strength and are summarised in Table 2 [19,24,25,26]. This had a total sample of n = 83 participants, with n = 70 men and n = 13 women. Two of the four studies demonstrated that high-intensity static stretching decreased strength more than lower intensity stretching [19,24]. Two studies measured both ROM and strength outcomes, therefore they appear in both Table 1 and Table 2. 

The studies demonstrated these findings in the hamstrings and quadriceps on an isokinetic dynamometer and stretched to 80, 100 and 120% of pre-intervention ROM [19] as well as up to 7/10 and 10/10 on a visual analogue scale [24]. One study measured the effects of different stretching intensities on strength immediately post eccentric muscle damage, with low-intensity stretching more beneficial in strength recovery than high-intensity stretching [25]. The last study compared stretching to 120% point of discomfort and the maximum point of discomfort and identified no change in peak torque; however, the angle at which this occurred was larger following the higher intensity of stretching [26].

### 3.3. Bias Assessment

The PEDro scores indicated that of the 18 studies included in this review, 15 studies were scored as high quality, three studies as moderate quality and zero studies as low quality (Table 3).

## 4. Discussion

This systematic review found that static stretching increases ROM, but it is inconclusive on whether high-intensity stretching leads to greater changes. Furthermore, the influence of static stretching intensity on strength outcomes are unclear. In this review, 18 studies were included. Of the 18 studies, 16 studies examined effects on ROM and four studies examining a strength outcome. Two studies measured both ROM and strength, therefore they were included in both sets of results in Table 1 and Table 2.

### 4.1. Static Stretching Intensity Effects on ROM 

This review examined outcomes from fourteen studies on the effects of different static stretching intensities on ROM, all of which observed an increase in ROM, regardless of the intensity used. Eight of the studies within this review demonstrated that higher intensity static stretching resulted in larger increases in ROM [19,26,29,30,31,32,37,41], the other eight studies found that there was no difference in the magnitude of change across differing intensities of stretching [27,33,34,35,36,38,39,40]. 

Acute increases in ROM after stretching are attributed to an increase in tolerance to the stretch [42], a decrease in MTU stiffness [43], alterations in musculotendinous visco-elasticity [19] and reflex activity [44]. All the studies within this review observed that ROM increased after static stretching, but only eight studies observed a greater increase in ROM from high-intensity static stretching versus lower intensity stretching. The results demonstrating greater ROM changes following higher intensity stretching may have resulted from of a decrease in H-reflex activity, which has been observed to decrease during a static stretch [45]. Furthermore, the decrease in reflex activity is proportional to the intensity of the stretch [46,47], so a higher stretch intensity could lead to a greater decrease in reflex activity and in turn increase ROM more.

Within the results of this review, there is heterogeneity in the methods and the outcomes. This is a result of the different methods used to define the threshold. For example, studies instruct participants to identify the “point before discomfort”, “point of mild discomfort”, “point of discomfort” and “point of pain onset”. In turn, to assist in future comparisons of studies, it would help if studies identified absolute (i.e., degree) and relative changes from a standardised non-stretching reference point. Future research can compare outcomes of stretching intensity based upon comparison to a reference point and include the total change in degrees from this point. In addition, humans can perceive and rank sensation from mechanical stimuli [48,49], therefore blinding of participants to experimental conditions is difficult and in turn, influences the bias assessment of this review as no studies scored in item five of the PEDro scale (blinding of subjects). Furthermore, as the stretching is delivered by an investigator, often using an isokinetic dynamometer which they operate, investigators will also not be blinded, with all studies also scoring zero on item six of the PEDro scale (blinding of researchers). All the studies scored for random allocation to study conditions; however, there was inadequate information provided by the studies on the process of participant randomisation to the study conditions. Therefore, future studies should use unblinded operators and then blinded data analysts. There is also scope for undertaking studies examining stretching intensity with participant deception to the intensity being used to prevent participants’ preconceived conceptions of pain or discomfort. 

Other differences may have resulted from the muscle group used. For example, some studies stretched the hamstring group within their experiment [19,26,27,29,30,31,33,35,36,38,39], the plantor flexors [32,34,41] or the quadriceps group [37,40]. As such, there are differences in contractile and non-contractile structures crossing the joint that will influence fascial length, pennation angle and elastic properties following different intensities of stretching. For example, the hamstrings muscle group comprises biarticular muscles, however the quadriceps muscle group comprises monoarticular (vastus lateralis, vastus medialis and vastus intermedialis) and biarticular muscles (rectus femoris). This might explain the differences, as Nakamura et al. [37] examined the effect of static stretching at 120, 100 and 80% ROM on the shear elastic modulus (i.e., stiffness) of the vastus lateralis, vastus medialis and rectus femoris and identified a decrease in the rectus femoris but no changes in the other muscles of the quadriceps group. 

A lot of the studies are also cross over designs, whereby participants undertake both a high- and low-intensity condition. Therefore, the reliability of sensation of stretch and pain could influence the results. This is especially true as temperature [50], muscle damage [51], age [52], and menstrual hormonal effects (i.e., menstrual cycle) [53] are associated with reduced ROM and flexibility. There are some data demonstrating good reliability on intensity of stretching sensation and ROM [54]; however, this needs further examination over multiple visits to determine the reliability of perception of stretching intensity, especially at a range of intensities and ranges of motion. Furthermore, women only made up 17.7% of the total sample of participants included in this review on ROM. Therefore, with the known effects of the menstrual cycle effects on ROM [53], future research must examine the effects of static stretching intensity in women. 

Based upon the findings from this review, it can be concluded that static stretching increases ROM. A higher intensity of stretching may increase ROM more so than lower intensity stretching; however, this is not consistent, and this review demonstrated no specific threshold for stretching intensity to induce greater changes. 

### 4.2. Static Stretching Intensity Effects on Strength

Four studies examined the effect of stretching intensity on a strength outcome and were included in the review. Two of the four studies demonstrated that high-intensity static stretching decreased strength more than lower intensity stretching [19,24]. Similar to the results on ROM, a variety of muscles, including the hamstrings and quadriceps, were examined. As there were few results on this topic it is impossible to draw conclusions on the outcomes of stretching intensity on the effects of muscle to be able to produce force, and this is needed for future research. 

Underlying mechanisms that attribute loss of maximal strength following static stretching includes increased compliance of the MTU that lowers MTU stiffness [22,54] and lower motor unit activation [55]. There are also suggestions that the duration of static stretching is an important factor for inducing acute strength loss [56]. Within this review, Takeuchi and Nakamura [26] used a single bout of 20 s of stretching and observed no effect in peak torque following all the stretching intensities. However, Rodrigues et al. [24] and Kataura et al. [19] used two sets of 30 s with a 30 s interval and 180 s of stretching, respectively, with both observing a decrease. Due to the limited number of studies, further research should be conducted examining the relationship between the duration of stretching, alongside the intensity of the stretching to identify which variable is more important for a subsequent strength decrease.

### 4.3. Strengths, Limitations, and Future Directions

To the authors’ knowledge, this is the first review to systematically review the effects of stretching intensity on ROM and strength. A further strength to this review is the inclusion of all studies without restricting sex, training status, muscles stretched, method of static stretching intensity and year. Despite the methods of this study, the authors recognise several limitations to this study. Firstly, there are a low number of studies examining the effects of static stretching intensity on ROM and strength. This likely reflects that this as a new topic of investigation as the oldest study included within this review was published in 2015. A meta-analysis was also not possible due to the inconsistencies in reporting a change in ROM (i.e., percentage change or degrees). 

A key consideration of interpreting the results from this review is that many of the studies included have low ecological validity due to stretching on an isokinetic dynamometer. While available in specialist rehabilitation settings, the availability and access are limited for the majority of athletic populations. Therefore, stretching will typically be undertaken in static, dynamic and assisted formats with basic equipment. Isokinetic dynamometers will give detailed analysis of force and range of motion, measured at a high frequency (i.e., Hz) but this is against a loss in applicability to applied settings implementing strength and conditioning programmes.

## 5. Conclusions

In conclusion, static stretching increases range of motion, but it is not clear if higher intensity stretching leads to larger increases in range of motion. For strength, the effect of stretching intensity is also unclear due to the limited amount of research. There are various methods used to measure the intensity of stretching and these are not directly comparable without reference to a standard starting point.

## Figures and Tables

**Table 1:** Summary of studies included in the systematic review that investigated the effect of static stretching intensity on range of motion (ROM) outcomes.

Study	n	Participants	Study Design	Muscles Stretched	Static Stretching Protocol	ROM Measure	Results	Comments
Freitas et al. [29]	17	Men (22.1 ± 2.7 years) from a university population.	CO, B	Hamstrings Supine knee extension on isokinetic dynamometer on right side.	1. High intensity at 100% of maximum tolerable passive torque with moderate duration (243.5 ± 69.5 s). 2. Low intensity at 50% of tolerable passive torque for long duration of 900 s	Absolute (°) knee extension maximal ROM at 1 min, 30 min and 60 min post stretching.	High-intensity, short-duration stretching increased ROM at 1, 30 and 60 min post stretching. No change for low intensity and high duration. Average intensity for high-intensity stretching was 107.3 ± 7.6% of initial maximal ROM versus 71.9 ± 4.2% for the low intensity.	High-intensity, short-duration stretching at 100% of maximum tolerable passive torque increases ROM, whereas there was no change following low-intensity, long-duration stretching.
Freitas et al. [30]	17	Men (23.9 ± 3.6 years), healthy with flexibility lower than 160° in an active knee extension test.	R, CO, B	Hamstrings. Passive knee extension, supine with 90° hip flexion on isokinetic dynamometer	Three separate laboratory visits: 180 s at 50% 135 s at 75% 90 s at 100% Intensity determined as a percentage of maximum tolerated stretch torque without pain. 5 repeats of each.	Peak angle of knee extension while supine	Peak angle changes pre vs. post stretching. 100%: 14.5 ± 11.2° * 75%: 4.0 ± 7.6° 50%: 1.8 ± 8.5° Passive Peak torque changes pre vs. post stretching: 100%: 19.8 ± 27.6 Nm * 75%: −3.4 ± 13.0 Nm 50%: −5.6 ± 15.9 Nm * Significant difference between pre versus post	High-intensity stretching for 90 s at 100% maximum tolerable torque increased peak joint angle and passive peak torque. However, 75 and 50% did not increase the angle or passive peak torque despite more time under stretch.
Fukaya et al. [31]	23	Men, (20.0 ± 1.5 years), healthy and not undertaking training, participants could not achieve full extension of knee with hip flexion at 110°.	Ra, P	Hamstrings. Passive knee extension on isokinetic dynamometer with hip and knee flexion starting at 110°	60 s of static stretching for 3 days per week for 4 weeks at 100% (n12) or 120% (n11) intensity. 100% defined as maximum tolerable ROM without pain.	ROM of knee extension from the initial position.	ROM Baseline 100% group 74.9 ± 9° 120% group 77.2 ± 5.7° 4 weeks 100% group 88.7 ± 6.9° * 120% group 90.0 ± 4.6° * * Significant difference from baseline	ROM increased from baseline for both 100% and 120% maximum tolerable ROM without pain, with no difference between the intensities.
Fukaya et al. [32]	18	Men (n = 11), women (n = 7), (21.5 ± 0.5 years), healthy and did not play sports or have high activity.	R, CO	Gastrocnemius. Seated in isokinetic dynamometer with 70° hip flexion.	120% for 100 s (high intensity, short duration) and 50% for 240 s (low intensity, long duration), 1 week apart. Intensity was based off 100% intensity as max dorsiflexion ROM to point of discomfort.	Dorsiflexion ROM	High-intensity ROM Pre: 26.0 ± 9.7 ° Post: 32.1 ± 11.2° *,# %change: 25.7 ± 19.9 Low intensity ROM Pre:24.5 ± 8.1 ° Post: 28.2 ± 8.5 * %change: 16.0 ± 11.8 * different pre vs. post # different high versus low	High-intensity, short-duration stretching and low-intensity, long-duration static stretching both increase dorsiflexion ROM; however, there was a greater increase following high-intensity, short-duration static stretching.
Kataura et al. [19]	18	Men (n = 9), women (n = 9), (20.6 ± 1.2 years), healthy college population. Excluded if undertaking training.	R, CO	Right side hamstrings. Sitting on isokinetic dynamometer at 111.2 ± 2.5° hip and 111.0 ± 1.7° flexion.	180 s at each intensity 80%, 100% and 120% of pre-intervention ROM value at onset of pain.	Seated knee extension ROM	ROM mean change 80%: −0.17 ± 3.75° 100%: 4.9 ± 3.5° *,$ 120%: 5.9 ± 4.4° *,$ * different pre vs. post $ different to 80%	There is a greater increase in ROM after stretching with a higher intensity.
Munjai et al. [33]	22	Women (20 ± 1 years), physically active and not undertaking resistance, aerobic or flexibility exercise in the previous 6 months.	RA, P	Hamstrings. Passive knee extension on isokinetic dynamometer with hip flexion at 120° and lower leg at 50° below horizontal.	Stretching to the point of discomfort (POD) or point of pain (POP). Eight sets with 15 s between sets (total time of 4 min)	Absolute (°) ROM change of angle during passive straight leg raise and absolute distance (cm) during sit and reach test, immediately following the stretching and 24-h post.	Straight leg raise—median (interquartile range) increase POP 6° (2–11.5°) * POD 4 (2–8.5°) * POP 24-h post 5° (0.5–11°) * POD 24-h post 3° (1–5.5°) * * different to baseline, but not between conditions Sit and reach test POP 1.5 cm (0.25–2.5cm) * POD 2.5 cm (1–3.25cm) * POP 24-h post 2 cm (NR) * POD 24-h post 3° (NR) * * different to baseline, but not conditions	No greater change in ROM from higher intensity stretching (POP) over lower intensity stretching (POD).
Oba et al. [34]	14	Men (22.9 ± 1.0 years), with no history of lower-limb injury or neuromuscular disease.	R, CO	Right side, plantar flexors on isokinetic dyna-mometer.	Control, 50%, 75%, 100% constant torque stretching at the maximum passive resistive torque measured in the first visit.	Absolute (°) dorsiflexion angle Pre and Post stretching.	Pre vs. Post Control 36.8 ± 6.2° vs. 37.5 ± 5.4° 50% 37.3 ± 6.8° vs. 37.8 ± 6.5° 75% 35.2 ± 5.3.5° vs. 39.2 ± 6.7° * 100% 37.2 ± 6.2° vs. 43.3 ± 6.4° * * different to pre values	Constant torque stretching at 75 and 100% increased ROM, whereas there was no increase from the lower intensity of 50%.
Marchetti et al. [35]	15	Men (27.5 ± 6.1 years), resistance trained with 6 ± 2 years training experience.	R, CO	Hamstrings. Supine, passive hip flexion maintaining knee extension.	50% POD 6 sets of 40 s, total volume 240 s. 85% POD 3 sets of 40 s, total volume 120 s. Point of discomfort.	Laying supine with passive hip flexion to maximal ROM.	ROM change 50% POD: pre: 98.5° ± 8.44°, post: 103.4 ± 9.2° (Δ4.6%, d = 0.55) * 85% POD: Pre: 96.9 ± 9.5°, post: 109.3 ± 8.4° (Δ11.42%, d = 1.33) * * different pre vs. post	Both 50 and 85% POD static hamstring stretching increased ROM. There was no significant difference between the 50 and 85% POD protocols.
Melo et al. [36]	41	Men. Amateur soccer players with limited 15° ROM limit for active knee extension. Allocated into four conditions 1. NS (23.8 ± 4.1 years), 2. CLS (24.7 ± 4.8 years), 3. MDL (24.7 ± 4.8 years), 4. PLS (22.8 ± 2.1 yrs).	P, RA	Hamstrings. Supine, passive stretch, hip flexion/knee extension on the non-dominant limb.	1. No Stretching (NS) 2. Comfort level stretching (CLS) 3. Mild discomfort level (MDL) 4. Pain level stretching (PLS) Three sets of 30 s stretching, three sessions per week until completing a total of 10 sessions.	Laying supine, active and passive knee extensions (AKE, PKE) with goniometer.	Active ROM: 1. NS Pre: 152.4 ± 6.0°, Post: 154.1 ± 6.0° *, Post 10th: 155.2 ± 6.2° * 2. CLS Pre: 153.1 ± 6.5°, Post: 155.5 ± 6.5° *, Post 10th: 156.4 ± 6.3° * 3. MDL Pre: 153.4 ± 5.6°, Post: 157.5 ± 6.6° *, Post 10th 164.2 ± 3.5° * 4. PLS Pre: 155.0 ± 5.6 °, Post: 159.2 ± 5.5° *, 167.6 ± 3.6° * Passive ROM: 1. NS Pre: 155.7 ± 5.6°, Post: 156.9 ± 6.7° *, Post 10th: 157.1 ± 6.0° * 2. CLS Pre: 155.7 ± 7.0°, Post: 160.5 ± 6.5° *, Post 10th: 158.3 ± 6.1° * 3. MDLS Pre: 156.5 ± 5.8°, Post: 160.6 ± 7.1° *, Post 10th: 166.1 ± 3.6° * 4. PLS Pre: 156.8 ± 5.7°, Post: 164.2 ± 5.5° * Post 10th: 170.2 ± 3.4° * * different to baseline	Passive static stretching for all intensities obtained an increase in active and passive knee extension ROM. There was also no difference in improvement of ROM following one session or ten stretching sessions.
Nakamura et al. [37]	18	Men (22.7 ± 2.8 years) healthy and sedentary.	R, CO	Quadriceps, knee flexion with 30 degrees hip extension on the dominant leg.	120%, 100%, 80% intensities based on knee flexion ROM at PRE-testing. Three 60 s stretching with 30 s rest.	Knee flexion ROM. 90° hip and knee flexion of non-dominant leg and 30° hip flexion of dominant leg as reference position. Investigator flexed the dominant leg to point of discomfort.	Pre versus post 120% and 100% increased, and 80% there was no change. Comparing outcomes of post knee flexion ROM, 120% was higher than 80%, but not different to 100%. The 100% condition was also not different to 80%. Data reported within figures.	Knee flexion ROM was increased following 120% and 100% intensities, with no change in ROM following a lower intensity of 80%.
Santos et al. [27]	20	Men (21.7 ± 2.5 years) untrained, university students with knee extension ROM of less than 150°	P, SB	Hamstrings, supine with hip flexion of the knee at 90°	Three 60 s stretching with 30 s rest. Submaximal intensity or maximal intensity Intensity based on numerical rating scale (NRS) of pain/discomfort (0–10). Low intensity between 1 and 2 and high between 9 and 10.	Seated knee extension on isokinetic dynamometer with thigh in 110° flexion. Researcher manually extended participants leg on the dynamometer to the initial perception of discomfort (ROMinitial) and then to maximum stretching limit (ROMmax).	Greater relative change (%) in Low intensity stretching for both ROMinitial and ROMmax Low intensity: Pre ROMinitial: 106.2 ± 13.2° (↑9.5%) Post ROMinitial: 116.3 ± 11.4° (↑4.6%) PreROMmax: 132.6 ± 13.3° PostROMmax: 138.7 ± 15.4° High intensity: Pre ROMinitial: 102.0 ± 13.5° Post ROMinitial: 110.7 ± 13.2° (↑8.5%) PreROMmax: 130.5 ± 15.9° PostROMmax: 133.4 ± 14.9° (↑2.2%)	There was an increase in the initial and maximum range of motion for both the intensities. However, there was a greater relative change (%) in the low-intensity stretching condition for both ROMinitial and ROMmax.
Takeuchi et al. [38]	12	Men (21 ± 0.8 years) recreationally active.	R, CO	Hamstrings, passive knee extension seated on isokinetic dynamometer with back angle at 60°.	60 s of static stretching (30 s stretching with 30 s rest) at 100% and 120% point of discomfort and (POD).	Passive knee extension ROM at 5 degrees per second immediately following the stretching.	Data reported within figures. Knee extension ROM increased in both intensities with no difference between the intensities.	Knee extension ROM is increased by both 100 and 120% stretching to the point of discomfort immediately following the stretching.
Takeuchi et al. [39]	14	Men (20.9 ± 0.7 years), healthy and physically active.	R, CO	Hamstrings, passive knee extension seated on isokinetic dynamometer with back angle at 60°.	60 s of static stretching (30 s stretching with 30 s rest) at 100% and 120% point of discomfort and (POD).	Passive knee extension ROM at 5 degrees per second. Performed pre-stretching, immediately post and 10 and 20 min post.	Knee extension ROM increased in both intensities with no difference between the intensities.	Knee extension ROM is increased by both 100 and 120% stretching to the point of discomfort immediately, 10 min and 20 min following the stretching.
Takeuchi and Nakamura [26]	13	Men (n = 9), (21.2 ± 0.4 years) women (n = 4), (21.3 ± 0.5 years) healthy but not training.	R, CO	Hamstrings, passive knee extension seated on isokinetic dynamometer with back angle at 60°.	20 s of static stretching at point of discomfort (POD), 120% POD and MaxPOD.	Passive knee extension ROM at 5 degrees per second immediately following the stretching.	ROM POD: Pre: 57.0 ± 14.0°, Post: 64.0 ± 13.6° *, %change 113.5 ± 10.4 120% POD: Pre: 57.3 ± 12.0°, Post: 71.8 ± 12.4° *, %change 127.6 ± 18.8 MaxPOD Pre: 57.0 ± 14.2°, Post: 75.3 ± 12.5° * †, %change 135.6 ± 18.5 * different to pre † different to post POD	ROM is increased following 20 s of static stretching at POD, 120% and MaxPOD. Stretching to the MaxPOD resulted in the largest change of hamstring ROM.
Takeuchi et al. [40] Experiment 1	11	Exp 1: 11 men (23.8 ± 3.4 years), healthy but not training.	R, CO	Quadriceps, knee flexion with 30 degrees hip extension on the dominant leg.	Three sets of 20 s (1 min) and three sets of 60 s (3 min) stretching at 120% ROM based on pre-intervention ROM value.	Knee flexion ROM. 90° hip and knee flexion of non-dominant leg and 30° hip flexion of dominant leg as reference position. Investigator flexed the dominant leg to point of discomfort.	ROM 1 min: Pre: 128.2 ± 9.2° Post: 145.9 ± 6.5° * 3 min Pre: 123.4 ± 11.4° Post: 136.8 ± 9.8° * * different to pre-condition.	Knee flexion ROM increased following stretching at 120% ROM, following 1 min and 3 min, but was not different for these durations of stretching.
Takeuchi et al. [40] Experiment 2	15	Exp 2: 15 men (23.1 ± 2.9 years), healthy but not training.	SI	Quadriceps, knee flexion with 30 degrees hip extension on the dominant leg.	Three sets of 60 s (3 min) stretching at 110%ROM based pre-intervention ROM value.	Knee flexion ROM. 90° hip and knee flexion of non-dominant leg and 30° hip flexion of dominant leg as reference position. Investigator flexed the dominant leg to point of discomfort.	ROM Pre: 128.7 ± 9.8° Post: 142.4 ± 7.8° * * different to pre-condition Compared with Exp 1, there was no difference on ROM between the intensities.	Knee flexion ROM increased following stretching at 110% ROM for 3 min. There was no difference between 120% ROM stretching for 1 and 3 min and stretching at 110% ROM for 3 min.
Nakamura et al. [41]	28	High intensity: 14 men (20.9 ± 0.5 years). Low intensity: 14 men (21.4 ± 1.0 years), healthy, sedentary.	RA, P	Gastrocnemius, passive reclined at 70° hip angle and 0° knee angle on isokinetic dynamometer.	Participant administered on 11-point verbal numerical scale of the dominant. 1. High intensity at 6–7 2. Low intensity at 0–1 Three sets for 60 s with 30 s intervals. Three days per week for four weeks.	Absolute (°) dorsiflexion on the non-dominant leg.	ROM High intensity Pre 16.5 ± 8.3° Post 21.9 ± 8.5° * Low intensity 20.1 ± 7.3° 21.5 ± 6.8° * Significant difference to pre	High-intensity stretching of the dominant leg increased dorsiflexion ROM in the contralateral joint (i.e., non-dominant leg), whereas low intensity stretching did not.

ROM, Range of Motion; P, Parallel Groups; CO, crossover; B, Balanced order of experimental conditions; R, Randomised order of conditions; RA, Random allocation to experimental conditions/groups; MBI, magnitude-based inference; SB, single-blind where the data analysis and the delivery of the stretching performed by different researchers; SI, single intervention study design with all participants undertaking one condition; NR, Not-reported.

**Table 2:** Summary of studies included in the systematic review that investigated the effect of static stretching intensity on strength.

Studies	N	Participants	Study Design	Muscles Stretched	Static Stretching Protocol	Strength Measure	Results	Comments
Apostolopoulos et al. [25]	30	Men (25 ± 6 years), actively involved in resistance training. Split into three groups of n10.	P, Ra	Hamstrings, hip flexors, Quadriceps, passive static stretching bilaterally.	1. Control (no stretching), 2. Low intensity (30–40% max perceived stretch) 3. High intensity (70–80% max perceived stretch) Max perceived stretch based upon 0–10 numerical rating scale. Three sets of 60 s stretching over 3 consecutive days following unaccustomed eccentric exercise.	Eccentric and isometric peak torque of right knee extensors 0, 24, 48 and 72 h post.	Eccentric peak torque (Nm) Low intensity—0 h 247.5 ± 62.0, 24 h 229.6 ± 62.8, 48 h 244.3 ± 55.3, 72h 263.1 ± 61.9. High intensity—0 h 218.2 ± 59.7, 24 h 173.4 ± 35.6, 48 h 208.0 ± 44.7, 72 h 195.9 ± 31.9. Control—0 h 214.8 ± 52.7, 24 h 196.2 ± 49.8, 48 h 179.4 ± 42.8 72 h 200.6 ± 65.6. MBI—low intensity stretching had “most likely, very likely or likely beneficial” effects at 0 h to 24 h and 0 h to 72 h compared to high intensity. Isometric peak torque (Nm). Low intensity—0 h 207.6 ± 40.2, 24 h 196.4 ± 46.2, 48 h 209.5 ± 47.0, 72 h 222.3 ± 47.9. High intensity—0 h 181.3 ± 41.2, 24 h 163.5 ± 41.7, 48 h 172.7 ± 50.1, 72 h 186.39.1. Control—0 h 185.1 ± 55.2, 24 h 161.5 ± 49.5, 48 h 169.6 ± 50.6, 72 h 172.8 ± 55.4. MBI—low intensity had possibly “trivial or beneficial” effects at 24 and 48 h compared to high intensity and “possibly beneficial or likely beneficial” at 48 and 72 h compared to control. MBI was “unclear” comparing low versus high at all time points.	Low intensity versus high-intensity passive static stretching is more beneficial on strength recovery following unaccustomed eccentric exercise.
Kataura et al. [19]	18	9 men, 9 women (20.6 ± 1.2 years), healthy college population. Excluded if undertaking training.	R, CO	Right side hamstrings. Sitting on isokinetic dynamometer at 111.2 ± 2.5° hip and 111.0 ± 1.7° flexion.	180 s at each intensity 80%, 100% and 120% of pre-intervention ROM value at onset of pain	Maximal isometric knee flexion for 6 s	Isometric muscle force mean change 80%: −1.2 ± 3.7 Nm 100%: −3.3 ± 5.1 Nm * 120%: −2.9 ± 5.9 Nm * * different pre vs. post	Isometric muscle force decreased significantly after stretching compared with before stretching at 100% and 120% intensities. However, there was no difference between the relative or absolute change for all stretch intensities.
Rodrigues et al. [24]	22	Men (24 ± 3 years), recreationally active and involved in more than 2 years resistance training.	R, CO	Quadriceps of dominant leg. Stood upright, one leg pulled to full knee flexion.	Visual analogue scale 0–10. Two sets of 30 s with 30 s rest interval. Submaximal at 7 Maximal at 10	Knee extension peak moment at 30°/s at ROM of 100°.	Concentric peak moment No stretching: 274.8 ± 13.39 Nm * Maximal intensity stretching: 246.0 ± 13.1 Nm Submaximal stretching—no change and data reported in a figure.	Stretching to an intensity of maximal discomfort produces a drop in strength when compared with no stretching or stretching to 70%.
Takeuchi and Nakamura [26]	13	9 Men (21.2 ± 0.4 years) 4 women (21.3 ± 0.5 years) healthy but not training.	R, CO	Hamstrings, passive knee extension seated on isokinetic dynamometer with back angle at 60°.	20 s of static stretching at point of discomfort (POD), 120% POD and MaxPOD.	Peak torque of knee flexion during maximum voluntary isokinetic concentric contraction at 60°/s and knee angle.	Peak torque % change POD: 99.1 ± 14.0 120% POD: 95.4 ± 17.4 MaxPOD: 98.4 ± 20.1 Angle at peak torque POD: 48.6 ± 10.9 120% POD:56.0 ± 19.9 * MaxPOD: 56.5 ± 17.2 *† * different to pre † different to post POD	There was no change in peak torque following all intensities of stretching, but the knee angle at peak torque at 120% POD and MaxPOD was different.

ROM, Range of Motion; P, Parallel Groups; CO, crossover; R, Randomised order of conditions; MBI Magnitude-based inference; POD Point of discomfort.

**Table 3:** PEDro scale scores of studies included within the systematic review.

	Eligibility Criteria Were Specified	Random Allocation	Concealed Allocation	Similar Baseline Characteristics	Blinding of All Subjects	Blinding of Researchers	Blinding of Assessors Measuring a Key Outcome	Outcome Measures Collected from a Minimum of 85% of Participants	Participants Were Tested as Planned within the Study Design	Results of Between-Group Statistical Comparisons Are Reported for at Least One Key Outcome	Measures of Variability for at Least One Key Outcome	Total
Freitas et al. [29]	1	1	0	1	0	0	0	1	1	1	1	6
Freitas et al. [30]	1	1	0	0	0	0	0	1	1	1	1	5
Fukaya et al. [31]	1	1	0	1	0	0	0	1	1	1	1	6
Fukaya et al. [32]	1	1	0	1	0	0	0	1	1	1	1	6
Kataura et al. [19]	1	1	0	1	0	0	0	1	1	1	1	6
Munjai et al. [33]	1	1	0	1	0	0	0	1	1	1	1	6
Oba et al. [34]	1	1	0	1	0	0	0	1	1	1	1	6
Marchetti et al. [35]	1	1	0	1	0	0	0	1	1	1	1	6
Melo et al. [36]	1	1	1	1	0	0	1	1	1	1	1	8
Nakamura et al. [37]	1	1	0	0	0	0	0	1	1	1	1	5
Santos et al. [27]	1	1	0	1	0	0	1	1	1	1	1	7
Takeuchi et al. [38]	1	1	0	1	0	0	0	1	1	1	1	6
Takeuchi et al. [39]	1	1	0	1	0	0	0	1	1	1	1	6
Takeuchi and Nakamura [26]	1	1	0	1	0	0	0	1	1	1	1	6
Takeuchi et al. 2021 [40]	1	1	0	1	0	0	0	1	1	1	1	6
Apostolopoulos et al. [25]	1	1	0	1	0	0	0	1	1	1	1	6
Rodrigues et al. [24]	1	1	0	0	0	0	0	1	1	1	1	5
Nakamura et al. [41]	1	1	0	1	0	0	0	1	1	1	1	6

## Data Availability

No new data was produced from this investigation.
